# Algorithmic bias in surgical risk prediction models and its impact on patient safety: a review

**DOI:** 10.1186/s13037-026-00495-x

**Published:** 2026-06-23

**Authors:** Mohamed Mustaf Ahmed

**Affiliations:** Faculty of Medicine and Health Sciences, SIMAD University, Mogadishu, Somalia

**Keywords:** Algorithmic bias, Surgical risk prediction, Machine learning, Health equity, Patient safety, Fairness in artificial intelligence

## Abstract

The increasing adoption of machine learning and artificial intelligence in surgical risk prediction has introduced new challenges related to the fairness and equity of these algorithms. These models range from regression-based risk calculators to machine learning systems, and differential performance may reflect poor calibration within a group, which is mainly a safety concern, or unequal performance between groups, which is mainly an equity concern. Predictive models trained on surgical registry data have shown differential performance across racial and ethnic groups in some studies, raising concerns about the potential for these tools to perpetuate or amplify existing disparities in surgical care. This review examines the sources and mechanisms of algorithmic bias in surgical risk prediction models, evaluates the evidence for differential model performance across patient subgroups, and discusses emerging debiasing strategies and regulatory frameworks. Training datasets from major surgical registries frequently contain incomplete or poorly granular race and ethnicity data, and the inclusion of race as a predictive variable remains controversial. Individual studies have reported lower sensitivity or higher false-negative rates for specific subgroups, potentially leading to an underestimation of surgical risk in those groups, although such discrimination-based measures do not by themselves establish miscalibration or demonstrated harm. Debiasing techniques, including reweighting, adversarial training, and fairness-aware multitask learning, have shown promise but remain largely untested in surgical contexts and are constrained by inherent trade-offs between within-group calibration and error rate parity across groups. Although regulatory bodies have begun to address algorithmic fairness, standardized auditing frameworks for surgical prediction models are still lacking. This review highlights the need for multicenter, demographically diverse validation studies, transparent model reporting, and equity-focused governance to ensure that artificial intelligence in surgery serves all patients safely and equitably.

## Clinical trial registration

Not applicable.

## Introduction

Machine learning and artificial intelligence have emerged as promising tools for predicting postoperative complications, stratifying surgical risks, and informing perioperative decision-making [1]. The appeal of these technologies lies in their capacity to analyze complex clinical data and generate individualized risk estimates that may surpass the accuracy of traditional scoring systems, such as the American Society of Anesthesiologists physical status classification [2]. Many of the surgical risk tools in routine clinical use, however, including the American College of Surgeons National Surgical Quality Improvement Program (ACS-NSQIP) Surgical Risk Calculator and the Physiological and Operative Severity Score for the enUmeration of Mortality and Morbidity, remain regression based models applied to structured tabular data rather than machine learning systems, so concerns about differential performance are not unique to newer machine learning methods [3]. As healthcare systems worldwide seek to improve surgical safety and reduce preventable adverse events, the development and validation of predictive algorithms have been accelerated. The extent of this integration should not be overstated, however, since a recent study of artificial intelligence models for preventing surgical complications found that, despite frequently strong reported accuracy, only a few have been adopted in routine clinical workflows, with implementation limited by usability, trust, workflow, financial, and ethical barriers [4].

However, the promise of algorithmic precision has been accompanied by a growing recognition, articulated in recent perspectives on medical artificial intelligence, that predictive models can compound existing inequities in healthcare across groups defined by race, ethnicity, and socioeconomic status [5]. Racial and ethnic disparities in surgical outcomes have been documented. For instance, African American patients undergoing elective spine surgery face significantly higher odds of readmission, non-routine discharge, and mortality than White patients [6]. Similarly, socioeconomic disadvantage is associated with lower completion of several enhanced recovery process measures and higher complication rates following colorectal surgery [7]. When predictive models are trained on datasets that reflect these pre-existing inequities, they may reproduce them, as demonstrated by a landmark analysis of a widely used population health algorithm that assigned lower risk scores to Black patients than to equally sick White patients because it predicted healthcare costs as a proxy for illness severity, so that the bias originated in the choice of prediction target rather than in the learning method itself [8]. Although this algorithm was not a surgical tool, the same problem could arise whenever surgical models were trained on outcome labels shaped by unequal access to care. These examples underscore how biased training data can undermine the safety goals of the technologies designed to advance them.

Because the term bias carries several distinct meanings, it is used in this review in a deliberately specific manner. Statistical bias refers to systematic errors in a model’s estimates, including miscalibration, in which predicted risks do not match observed outcomes. Sampling or representational bias refers to training data that do not reflect the target population of interest. Social or discriminatory bias refers to model behavior that produces unjustified differences between protected groups. Methodological risk of bias, as assessed by instruments such as the Prediction model Risk of Bias Assessment Tool (PROBAST), refers to flaws in study design, conduct, and analysis [9]. Throughout this review, each claim about bias is linked to whichever of these meanings applies, and a distinction is maintained between safety, understood as agreement between predicted and observed risk within a group [10], and equity, understood as the distribution of model performance and benefit between groups. A model can be well calibrated within every group yet distribute benefit unequally, and it can appear equitable on aggregate discrimination metrics such as the area under the receiver operating characteristic curve (AUROC) yet be poorly calibrated, so the two properties require separate evaluation and separate remedies [10, 11].

This problem is aggravated by the opaqueness of many machine learning architectures. Complex ensemble methods and deep neural networks often function as opaque decision systems, making it difficult for clinicians to identify when and how bias enters the prediction pipeline [12, 13]. Furthermore, surgical registries and institutional databases that serve as training data for these models frequently suffer from incomplete demographic reporting and underrepresentation of smaller and community hospitals [14]. A scoping review of racial bias in clinical machine learning found that 67% of the included studies concluded that racial bias was present in the evaluated models; however, fairness metrics were applied inconsistently across the included studies [12].

To our knowledge, no comprehensive review has specifically examined algorithmic bias in surgical risk prediction models and its implications for patient safety and equity. While existing reviews have broadly addressed artificial intelligence in surgery or racial bias in healthcare algorithms, the intersection of these domains, where biased predictions directly influence perioperative resource allocation, informed consent, and clinical decision-making, remains unexplored. A recent review, for example, synthesised evidence that artificial intelligence and machine learning can support patient safety across the preoperative, intraoperative, and postoperative phases of complex surgery, with machine learning models reported to outperform traditional risk scores and computer vision supporting the verification of safety-critical operative steps [15]. Such reviews have generally emphasized aggregate performance and overall safety gains rather than how predictive accuracy and benefits are distributed across racial, ethnic, and socioeconomic subgroups, which is the focus of the present review. This review aims to provide a focused overview of the mechanisms, evidence, and mitigation strategies for algorithmic bias in surgical risk prediction, with particular attention to its consequences for ensuring equitable patient safety.

## Historical context of disparities in surgical outcomes

Disparities in surgical care along racial, ethnic, and socioeconomic lines have been recognized for decades. The Institute of Medicine’s 2003 report, Unequal Treatment, documented that Black patients in the United States received fewer clinically necessary procedures and poorer quality of care than White patients, independent of insurance status and clinical severity [16]. Subsequent research has consistently demonstrated that these disparities extend to several surgical specialties. A systematic review of over 5.5 million patients undergoing elective spine surgery found that African American patients had significantly increased odds of 90-day readmission (odds ratio [OR], 1.33), non-routine discharge (OR, 1.71), and mortality (OR, 1.66) compared with White patients [6].

In colorectal surgery, low socioeconomic status is associated with lower rates of bowel preparation completion, reduced electronic patient portal engagement, and longer median length of stay [7]. Black patients in the same cohort demonstrated increased odds of respiratory complications, with an odds ratio of 4.11, despite high overall rates of enhanced recovery protocol compliance [7]. These disparities are thought to arise from a complex interplay of factors, including differential access to surgical care, implicit clinician bias, structural racism, and socioeconomic health determinants. When machine learning models are trained on data generated within these inequitable systems, they risk encoding these disparities as predictive features rather than recognizing them as correctable injustices. These outcome disparities reflect inequities in the care system itself rather than errors introduced by any algorithm, a distinction that becomes important when interpreting model behavior later in this review.

## The machine learning pipeline in surgical risk prediction

Understanding how bias enters surgical prediction models requires examining the complete prediction modelling pipeline, which applies to both regression-based calculators and machine learning models, from data collection to clinical deployment. The process typically begins with the extraction of training data from surgical registries or electronic health record systems [17]. Major registries, such as the ACS-NSQIP, whose participant use files contain more than 12.5 million surgical cases [18], provide rich clinical datasets but typically underrepresent smaller and community hospitals [14]. Feature selection follows, during which investigators choose the patient characteristics, clinical variables, and contextual factors to be included in the model. Model development involves training algorithms on historical data and evaluating their performance using metrics such as AUROC, sensitivity, and specificity.

Each stage introduces opportunities for bias. At the data collection stage, training datasets may underrepresent minority patients and therefore fail to reflect the demographic diversity of surgical populations [19]. At the feature selection stage, the inclusion of race as a variable remains controversial, as it may serve as a proxy for unmeasured social determinants rather than a biologically meaningful predictor [20]. At the validation stage, a systematic review of 103 machine learning studies in perioperative medicine found that 75% relied on single-center validation, and only 13% (14 of 103 studies) were externally validated across multiple centers [1].

## Evidence of differential model performance across racial and ethnic groups

Several studies have indicated that prediction models for surgical risk can perform differently across racial and ethnic subgroups, although this evidence requires careful interpretation. Lucas et al. compared four algorithms, including a conventional logistic regression (LR) model alongside a multilayer perceptron (MLP), a random forest (RF), and an XGBoost (XGB) model, for predicting 30-day readmission after colorectal cancer resection using data from 112,077 patients in the ACS-NSQIP database [14]. They reported statistically significant differences in the AUROC, false-positive rate (FPR), and false-negative rate (FNR) between the racial groups across all models. To assess fairness, the authors applied the disparate impact, or four-fifths, rule, an administrative screening test that originated in United States employment discrimination law, under which a model is flagged for potential adverse impact when the ratio of favourable prediction rates between two groups falls outside a band of approximately 0.80 to 1.25 [14].

By this test, the models were fair for Black compared with White patients and failed the fairness criterion only for the heterogeneous ‘Other’ race category, which comprised less than 6% of the cohort and which the authors note may have included patients whose race was misclassified or not self-identified [14]. Among the four algorithms, MLP showed the best fairness, whereas LR, RF, and XGB models showed larger disparities. These are discrimination and error-rate measures rather than calibration, the disparate-impact finding was confined to a small and possibly miscategorized group, and the authors cautioned that their results may be confounded by factors not captured in the registry, such as social determinants of health and hospital or surgeon characteristics. For these reasons, the findings are best read as a signal of potential rather than demonstrated inequity, and the authors themselves conclude only that the models may perform unequally if used to direct care [14].

In a separate study, Hopkins et al. developed a deep neural network to predict surgical site infections (SSI) after posterior spinal fusion in 4,046 patients [21]. Their analysis of model weights revealed that the White race was identified as a protective factor against infection, raising questions about whether the model had learned genuine biological relationships or had captured patterns of differential care quality and access, an example in which a model appears to encode an upstream disparity rather than create one. A landmark study by Gichoya et al. demonstrated that deep learning models could predict patient self-reported race from medical images with an AUROC of 0.81 to 0.99 across multiple imaging modalities, including 0.91 to 0.99 for X-ray imaging, 0.87 to 0.96 for chest computed tomography, and 0.81 for mammography, even from corrupted or cropped images, through mechanisms that remain unexplained (Table 1) [22].

This finding suggests that race-related information may permeate medical data in ways that are invisible to human reviewers but detectable by algorithms, creating pathways for bias that are difficult to identify and control in the medical field. Because race can persist as a latent property of the data that is recoverable even when it is not an explicit input, it substantially complicates debiasing attempts. This evidence comes from diagnostic imaging rather than tabular surgical risk models and is included here as a mechanism that may extend to surgical data rather than as direct evidence of bias in a surgical risk score. Recent surgical applications have continued to emphasise overall predictive accuracy rather than subgroup fairness; for example, a 2026 machine learning model for SSI after major abdominal surgery compared four algorithms and reported a high AUROC of 0.934 for the best performing model, without assessing racial or ethnic subgroup fairness [23].

**Table 1:** Selected studies documenting algorithmic bias in surgical and perioperative prediction models

Surgical domain	Algorithm	Bias metric used	Key finding	Source
Colorectal resection	LR, MLP, RF, XGB	FNR and FPR differences, disparate impact	Significant differences in AUROC, FPR, and FNR across all models; by the four-fifths rule, only the small ‘Other’ category failed the fairness test, and MLP was the fairest model	[14]
Perioperative general	Multiple ML models	PROBAST risk of bias	Single-center validation in 75% and external validation in only 13%; high or unclear risk of bias in 90%	[1]
Medical imaging, multiple	Convolutional neural network	AUROC for race prediction	Deep learning predicted race from imaging with AUROC of 0.81 to 0.99 across modalities, mechanism unknown	[22]
Posterior spinal fusion	Deep neural network	Variable weight analysis	White race identified as protective factor against SSI in model weights	[21]
Clinical ML general	Various ML	Equal opportunity, disparate impact	67% of studies concluded racial bias was present in models	[12]

## The controversy of race as a variable in surgical prediction

The role of race in clinical prediction models has become one of the most debated topics in terms of algorithmic fairness. Race is a social construct without consistent biological underpinning; however, it correlates with numerous health outcomes due to its relationship with systemic factors, including racism and experiences of discrimination, socioeconomic status, and broader social and environmental conditions [20]. When included as a predictor in surgical risk models, race may serve as a proxy for these unmeasured social determinants, leading to predictions that reflect structural inequities rather than intrinsic patient risks.

Several prominent clinical tools have historically included race-based adjustments in their development. The estimated glomerular filtration rate formula, vaginal birth after cesarean calculator, and certain cardiac risk scores have all incorporated race as a variable, prompting vigorous debate about whether doing so improves prediction or perpetuates bias [20]. In surgery, this question is particularly consequential because biased risk stratification models may misclassify patients from certain racial groups, leading to disparities in resource allocation and post-operative care delivery. A higher FNR for some subgroups could mean that patients at risk of adverse outcomes are not identified for timely intervention, whereas differences in FPR could alter the balance of unnecessary interventions in the opposite direction. However, the direction and size of these effects varied by group and model in the available evidence, and in the colorectal readmission analysis, the ‘Other’ group showed the lowest FPR.

Addressing this requires extending model evaluation beyond aggregate performance metrics to include demographic subgroup analyses, incorporating fairness metrics such as disparate impact, FNR differences, and differential AUROC [14], together with calibration assessed within each subgroup, extending the principle that discrimination metrics alone cannot reveal whether predicted risks match observed outcomes [10], and augmenting models with social determinants of health, whose absence from registry data has been acknowledged as a potential source of unmeasured bias [14].

## Training data limitations and representational gaps in surgical registries

The quality and representativeness of the training data fundamentally determine the fairness of the surgical prediction models. Major surgical databases that serve as the foundation for most model developments suffer from several structural limitations (Table 2). Although the ACS-NSQIP contains millions of cases, it typically underrepresents smaller and community hospitals [14], and minority and uninsured patients have been shown to be substantially less likely to receive care at large academic medical centers [24]. Although the National Inpatient Sample (NIS) is broader in scope, it relies on administrative coding that captures limited clinical nuances and suffers from variable completeness of racial and ethnic data across states, with the race data element removed entirely from the 2023 release [25].

**Table 2:** Major surgical registries and databases used for training prediction models, with demographic data availability

Registry or database	Surgical scope	Approximate size	Racial and ethnic data	Known limitation	Source
ACS-NSQIP	Multi-specialty surgical	Over 12.5 million cases	Race recorded but often incomplete	Underrepresents smaller and community hospitals	[18]
NIS, HCUP	All inpatient procedures	Over 33 million weighted discharges per year	Race recorded historically; race element removed from the 2023 release	Administrative data, limited clinical detail	[25]
MIMIC-IV	ICU, emergency department, and general hospital inpatients	546,028 hospital admissions, 364,627 patients	Self-reported race and ethnicity included	Single institution, limited generalizability	[26]
UK National Joint Registry	Joint replacement	Over 4.5 million procedures	Ethnicity ascertained via HES linkage; minority ethnic groups underrepresented	Ethnicity not recorded directly; requires HES linkage	[27]
SEER-Medicare	Cancer surgery	Over 6 million cancer cases (1999–2021)	Race and ethnicity from cancer registries	Limited to Medicare-eligible population, age 65 and older	[28]

**Fig. 1:**
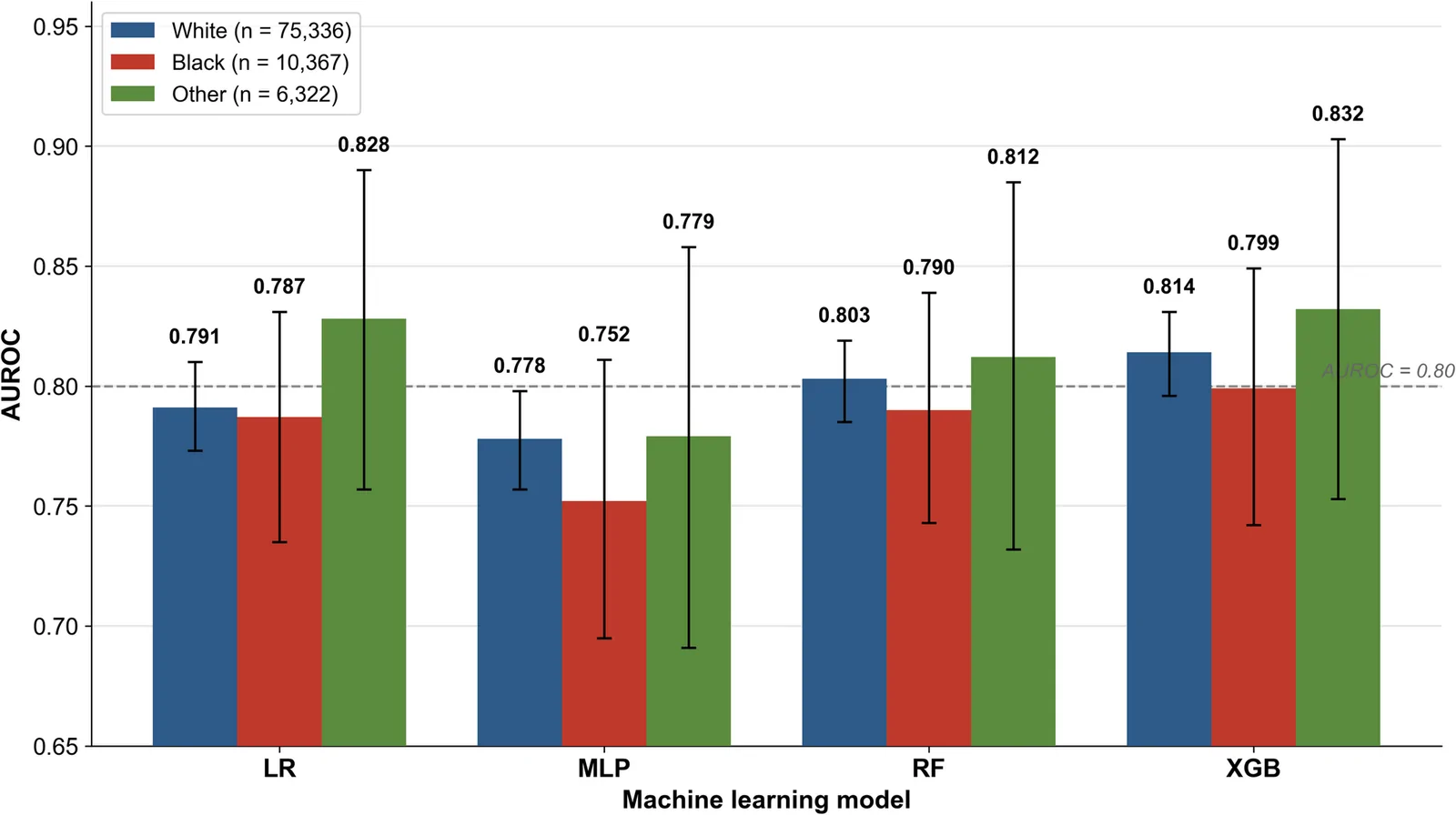
Differential AUROC by race in four prediction models for 30-day readmission prediction after colorectal cancer resection (n = 112,077 patients, ACS-NSQIP 2012 to 2020) [14]. Error bars represent the 95% confidence interval from bootstrap resampling (1,000 samples). The figure shows discrimination, measured by AUROC, which is a different property from the separate disparate-impact analysis reported by the same study; by that separate four-fifths-rule test, only the small ‘Other’ category fell outside the fairness band

Racial and ethnic data across the United States healthcare databases from which surgical models draw are frequently incomplete or inconsistently categorized, with the highest levels of misclassification among Asian, Pacific Islander, American Indian, and Hispanic patients [29]. The use of broad categories such as ‘Other’ or ‘Unknown’ can further obscure the diversity within and between minority populations [30]. International surgical registries present additional challenges, as racial and ethnic categorization schemes vary across countries, and some registries do not directly record ethnicity. For example, ethnicity in the National Joint Registry of England, Wales, and Northern Ireland has been ascertained through linkage to Hospital Episode Statistics (HES) rather than recorded by the registry itself, and analyses using this approach have documented substantially lower rates of hip and knee replacement among Black and Asian patients in England [31]. These representational gaps mean that models trained on registry data may perform well for well-represented populations while failing to achieve acceptable accuracy for underrepresented groups, with direct consequences for patient safety (Fig. 1).

## Debiasing strategies and fairness-aware machine learning

A range of debiasing techniques have been developed to mitigate algorithmic bias, which are generally categorized as pre-processing, in-processing, and post-processing methods (Table 3). These techniques are not a single general solution, and their suitability depends on the specific mechanism that produces a given disparity, as discussed below. Preprocessing approaches modify the training data before model development. Reweighing, a widely used pre-processing technique, assigns differential weights to training samples so that group membership becomes statistically independent of the outcome label, and it has demonstrated improvements in fairness metrics when applied to healthcare classification models [32]. In a study comparing multiple debiasing approaches, the Random Forest classifier augmented with reweighing achieved an accuracy of 88% and the most balanced fairness profile among the tested models, although its disparate impact score of 0.52 remained below the four-fifths benchmark described earlier.

In-processing methods directly integrate fairness constraints into model training objectives. Adversarial debiasing uses a secondary network that penalizes the primary model for using protected attributes; however, this approach increases architectural complexity and may not consistently improve all fairness metrics simultaneously [32]. A promising recent development is the FAIR-MTL framework, which integrates sensitive set invariance into the multitask learning architecture. When applied to predicting post-spinal fusion complications, this framework achieved an AUROC of 0.86 and an accuracy of 75% while reducing the demographic parity difference to 0.055 for gender and to 0.056 for age, although gender and age were the only attributes evaluated and race and ethnicity were not assessed, leaving the relevance of this framework to racial and ethnic fairness in surgery untested [33]. Post-processing methods, such as calibrated equalized odds, modify the predictions after model training, for example by mixing them with a group-specific base-rate classifier, to equalize an error rate across groups [34]. Although computationally simpler, these approaches require separate calibration datasets and may not address the root causes of bias within the model. However, these methods also face fundamental mathematical limitations. When outcome base rates differ across groups, a model cannot be simultaneously well calibrated within each group and satisfy equalized odds across groups unless it predicts perfectly, so calibration is compatible with at most a single error-rate constraint [34, 35].

**Fig. 2:**
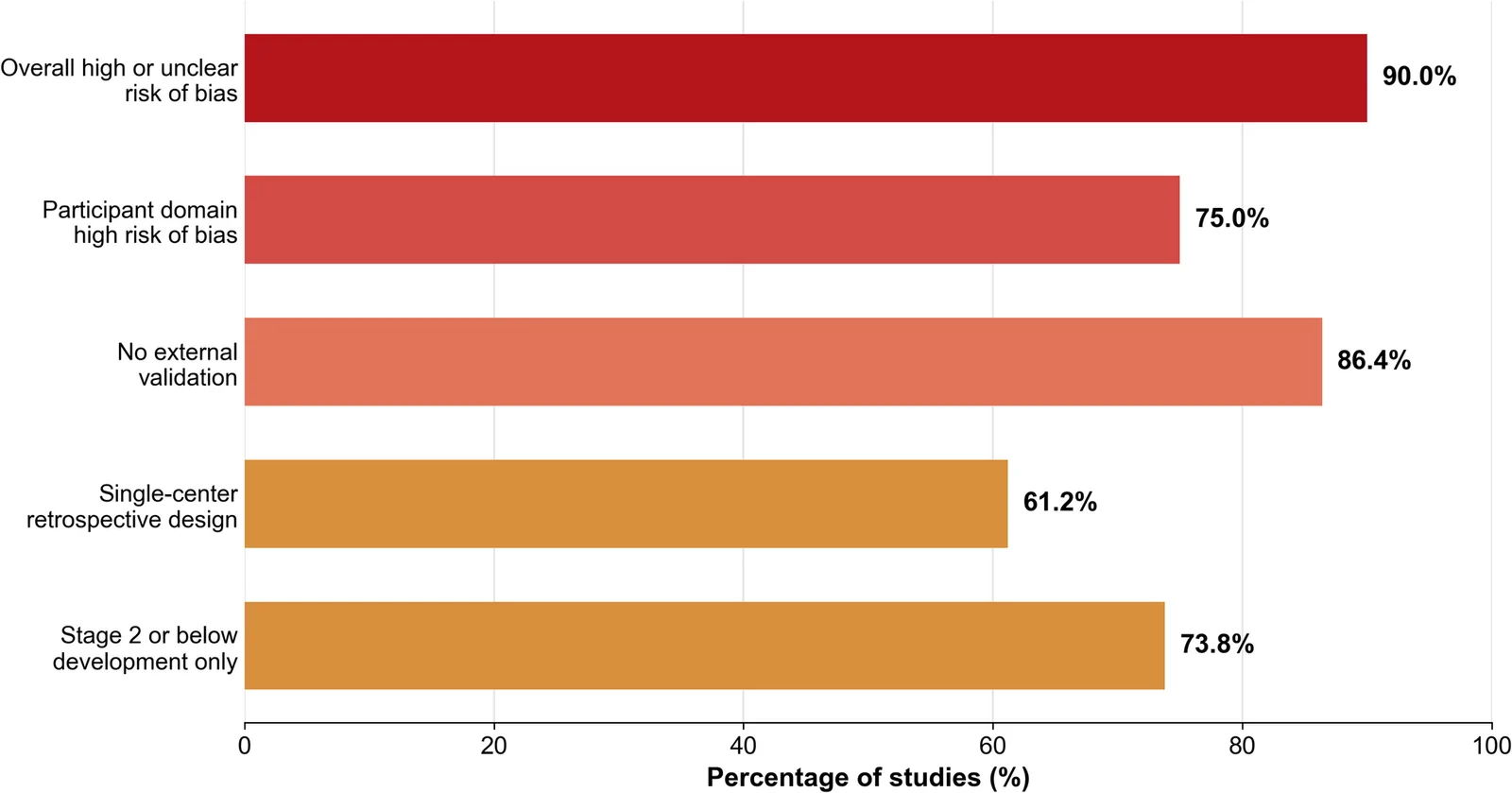
Quality and bias assessment of 103 perioperative machine learning studies using PROBAST [1]. The figure highlights the pervasive methodological limitations that create pathways for algorithmic bias, including a high or unclear overall risk of bias (90%), high risk of bias in the participant domain (75%), and near-universal absence of external validation (86.4%)

The post-processing solution analyzed by Pleiss et al. attains parity only by deliberately degrading the better performing group’s predictions, in part by withholding information on a randomly selected subset of patients, which the authors regard as unsatisfactory in high-stakes settings, and in one of their own test cases the constraint could not be satisfied at all [34]. Therefore, debiasing is not a simple correction that developers have neglected but a process governed by genuine and sometimes irreducible trade-offs. When a disparity originates upstream, in the training data, the outcome labels, or differing base rates rather than in the model itself, adjusting the model may fail to remove the disparity and can even degrade calibration, so the more appropriate response may be to improve the data and the choice of outcome label rather than to manipulate model outputs [8]. These debiasing strategies must also be weighed against the pervasive quality and bias issues identified in the perioperative machine learning literature (Fig. 2).

**Table 3:** Debiasing techniques for algorithmic fairness in healthcare AI, with applicability to surgical risk prediction

Debiasing technique	Category	Mechanism	Fairness improvement	Trade-off	Source
Reweighing	Pre-processing	Assigns weights to training samples to equalize group representation	Disparate impact score improved to 0.52	Accuracy increased with reweighing (76.68% to 88.01%), no trade-off observed in this study	[32]
Adversarial debiasing	In-processing	Adversarial network penalizes model for using protected attributes	Disparate impact score improved to 0.76; average odds difference improved to -0.16	Significant accuracy decrease (76.68% to 59.97%); increased model complexity	[32]
Exponentiated gradient	In-processing	Iteratively optimizes fairness constraints during training	Disparate impact score improved modestly (0.28 to 0.34); moderate fairness gain	Slight accuracy decrease (76.68% to 75.53%); weakest fairness improvement among tested methods	[32]
FAIR-MTL framework	Hybrid pre- and in-processing	K-means subgroup inference with multi-task learning and task-specific heads per subgroup	Demographic parity difference reduced to 0.055 (gender) and 0.056 (age); equalized odds difference 0.094 (gender) and 0.148 (age); not evaluated for race or ethnicity	Slight accuracy decrease (75% vs. 77%); requires task-specific architecture design	[33]
Calibrated equalized odds	Post-processing	Mixes predictions with a trivial classifier per group to preserve calibration with a single error constraint	Achieves equal false-negative or false-positive rates per group; full equalized odds is mathematically incompatible with calibration when group base rates differ	Coin-flip randomization of individual predictions; unsatisfactory in high-stakes clinical settings	[34]

## Regulatory landscape and governance frameworks for algorithmic fairness

Regulatory attention to algorithmic fairness in healthcare has increased in recent years, although comprehensive frameworks specific to surgical prediction models are still being developed. In the United States, an analysis of 168 machine learning-enabled medical devices authorised by the Food and Drug Administration in 2024 found that only a minority provided transparency and performance information, underscoring the need for stronger real-world performance monitoring and post-market surveillance [36]. The European Union’s Artificial Intelligence Act, adopted in 2024, classifies many healthcare AI systems, including medical-device software, as high-risk and mandates conformity assessments, data governance requirements, and human oversight provisions [37]. However, neither framework specifies auditing standards for demographic fairness in surgical prediction [37].

Professional surgical communities have begun to address this issue. Members of the American College of Surgeons Health Information Technology Committee have called for surgeon led, responsible adoption of artificial intelligence [38], and the call for algorithmic fairness has been echoed in specialty-specific literature, including orthodontics and craniofacial health [39]. Several frameworks for the ethical use of artificial intelligence in surgery have been proposed; however, translating their principles into enforceable standards with measurable compliance criteria remains a significant challenge. The absence of mandatory bias auditing for surgical prediction tools means that biased algorithms can enter clinical practice without a systematic evaluation of their implications for equity.

## Implications for informed consent and shared decision-making

Algorithmic bias also has direct implications for the informed consent process. When predictive models are miscalibrated for a subgroup, for example by underestimating the complication risk, the risk information communicated during preoperative consultations may be inaccurate, potentially leading patients to consent to procedures without a full understanding of their individualized risk profile [17]. Conversely, if models overestimate the risk for certain populations, patients may be inappropriately discouraged from undergoing beneficial surgical intervention.

The opacity of many machine learning models complicates these dynamics. Unlike traditional risk calculators, which produce interpretable outputs based on known clinical variables, complex ensemble and deep learning models may generate predictions that clinicians cannot readily explain to patients [13, 17]. This lack of transparency is particularly concerning when predictions vary systematically by demographic group, as neither the clinician nor the patient may be aware that the estimate is differentially calibrated. Ensuring that AI-assisted risk communication is both accurate and equitable requires rigorous subgroup validation of prediction models and education of clinicians regarding the limitations and potential biases of the tools they use.

## Toward equitable surgical artificial intelligence, recommendations for research and practice

Addressing algorithmic bias in surgical risk prediction requires coordinated action across multiple domains. First, the development of more representative and inclusive training datasets is essential. This requires critically analyzing whether existing datasets adequately represent minority patients [19] and improving the completeness and granularity of demographic data collection. Complementary measures include expanding surgical registry participation to community and safety-net hospitals and fostering multi-institutional collaborations that capture the full diversity of surgical populations. Federated learning approaches, which allow models to be trained across institutions without centralizing patient data, offer a promising pathway for increasing demographic diversity while preserving patient privacy [40].

Second, standardized fairness reporting is required for the development of surgical prediction models. Just as the Transparent Reporting of a multivariable prediction model for Individual Prognosis Or Diagnosis (TRIPOD) statement standardises the reporting of prediction models, analogous standards for demographic fairness assessment, including stratified performance metrics, fairness indices, and subgroup calibration data such as the calibration slope, the calibration intercept, and flexible calibration curves estimated within each demographic subgroup, should be incorporated into publication and regulatory review processes [1, 10]. Third, investment in fairness-aware machine learning research specific to surgical contexts is urgently needed. Although debiasing techniques have been developed and tested in other domains, fairness considerations remain largely unaddressed in surgical prediction models [33], and the suitability of these techniques for characteristics common in surgical data, such as rare outcome events, complex feature interactions, and temporal dependencies, requires a dedicated evaluation. Finally, surgical education and training programs should incorporate curricula on algorithmic literacy, enabling the next generation of surgeons to critically evaluate and responsibly deploy AI-based decision support tools in their practice. These recommendations face substantial practical barriers, including the limited adoption of such tools in routine practice to date, competing institutional priorities, the cost and complexity of multi-institutional data sharing, and the unresolved methodological trade-offs described above, so progress is likely to be incremental rather than immediate [4].

## Conclusion

Algorithmic bias in surgical risk prediction models represents an important and under-examined challenge for patient safety and equity. As predictive tools are increasingly considered for perioperative workflows, the potential for biased predictions to influence surgical decision-making, resource allocation, and informed consent deserves careful consideration. The evidence reviewed in this article indicates that some prediction models can exhibit differential performance across racial, ethnic, and socioeconomic groups, although much of this evidence rests on discrimination metrics and small or heterogeneous subgroups, and direct demonstrations of resulting harm remain limited. Many of the disparities that models display may originate upstream, in inequitable care systems, and in the data and labels they produce, rather than in the model architecture itself, a distinction that shapes which remedies are appropriate. These patterns also reflect the structural limitations of the training data, the controversial use of race as a predictive variable, and insufficient validation across diverse patient populations. Although promising debiasing techniques and regulatory frameworks are emerging, they are constrained by genuine methodological trade-offs, and their application in surgical contexts remains limited and largely untested in practice. Achieving equitable surgical artificial intelligence will require sustained and coordinated efforts spanning more inclusive data collection, standardized fairness auditing, context-specific research, and algorithmic literacy among surgical professionals. Progress is likely to be gradual; however, with careful attention to both safety and equity, predictive technologies can be developed and evaluated to serve all surgical patients more reliably, regardless of their demographic backgrounds.

## Data Availability

No datasets were generated or analysed during the current study.

## Declarations

### Declaration of AI use

The author acknowledges the use of Paperpal AI (https://paperpal.com/) for its "Language Edit" and "Make Academic" features to improve the clarity and readability of this manuscript. This assistance was limited to language refinement. All analyses and interpretations were solely performed by the authors.

## References

[1] Arina P, Kaczorek MR, Hofmaenner DA, Pisciotta W, Refinetti P, Singer M, Mazomenos EB, Whittle J. Prediction of complications and prognostication in perioperative medicine: A Systematic Review and PROBAST assessment of machine learning tools. Anesthesiology. 2024;140:85–101. 10.1097/ALN.0000000000004764. 37944114 PMC11146190

[2] Arvind V, Kim JS, Oermann EK, Kaji D, Cho SK. Predicting surgical complications in adult patients undergoing anterior cervical discectomy and fusion using machine learning. Neurospine. 2018;15:329–37. 10.14245/ns.1836248.124. 30554505 PMC6347343

[3] Hassan AM, Rajesh A, Asaad M, Nelson JA, Coert JH, Mehrara BJ, Butler CE. Artificial intelligence and machine learning in prediction of surgical complications: Current state, applications, and implications. Am Surg. 2023;89:25–30. 10.1177/00031348221101488. 35562124 PMC9653510

[4] Mevik K, Woldaregay AZ, Jonsson EL, Tejedor M, Temple-Oberle C. Application of AI models for preventing surgical complications: Scoping review of clinical readiness and barriers to implementation. JMIR AI. 2026;5:e75064. 10.2196/75064. 41701931 PMC12912657

[5] Mittermaier M, Raza MM, Kvedar JC. Bias in AI-based models for medical applications: challenges and mitigation strategies. NPJ Digit Med. 2023;6:113. 10.1038/s41746-023-00858-z. 37311802 PMC10264403

[6] Akosman I, Kumar N, Mortenson R, Lans A, De La Garza Ramos R, Eleswarapu A, Yassari R, Fourman MS. Racial differences in perioperative complications, readmissions, and mortalities after elective spine surgery in the United States: A systematic Review using AI-assisted bibliometric analysis. Global Spine J. 2024;14:750–66. 10.1177/21925682231186759. 37363960 PMC10802512

[7] Antoniv M, Maldonado LJ, Nikiforchin A, Gershanik EF, Bleday R. Enhanced Recovery After Surgery protocol compliance and early outcomes for elective colorectal procedures by race/ethnicity and socioeconomic status. Ann Surg. 2025. 10.1097/SLA.0000000000006622. 39781757

[8] Obermeyer Z, Powers B, Vogeli C, Mullainathan S. Dissecting racial bias in an algorithm used to manage the health of populations. Science. 2019;366:447–53. 10.1126/science.aax2342. 31649194

[9] Wolff RF, Moons KGM, Riley RD, Whiting PF, Westwood M, Collins GS, Reitsma JB, Kleijnen J, Mallett S, Group† P. PROBAST: A tool to assess the risk of bias and applicability of prediction model studies. Ann Intern Med. 2019;170:51–8. 10.7326/M18-1376. 30596875

[10] Van Calster B, McLernon DJ, van Smeden M, Wynants L, Steyerberg EW. initiative TG ‘Evaluating diagnostic tests and prediction models’ of the S. Calibration: the Achilles heel of predictive analytics. BMC Med. 2019;17:230. 10.1186/s12916-019-1466-7. 31842878 PMC6912996

[11] Paulus JK, Kent DM. Predictably unequal: understanding and addressing concerns that algorithmic clinical prediction may increase health disparities. NPJ Digit Med. 2020;3:99. 10.1038/s41746-020-0304-9. 32821854 PMC7393367

[12] Huang J, Galal G, Etemadi M, Vaidyanathan M. Evaluation and mitigation of racial bias in clinical machine learning models: Scoping review. JMIR Med Inf. 2022;10:e36388. 10.2196/36388. PMC9198828 35639450

[13] Anand K, Hong S, Anand K, Hendrix J. Machine learning: implications and applications for ambulatory anesthesia. Curr Opin Anaesthesiol. 2024;37:619–23. 10.1097/ACO.0000000000001410. 38979675 PMC11556868

[14] Lucas MM, Schootman M, Laryea JA, Orcutt ST, Li C, Ying J, Rumpel JA, Yang CC. Bias in prediction models to identify patients with colorectal cancer at high risk for readmission after resection. JCO Clin Cancer Inf. 2024;8:e2300194. 10.1200/CCI.23.00194. PMC11741203 39831110

[15] Ahmed MM, Othman ZK, Adebayo UO, Kasimieh O, Okesanya OJ, Musa SS, Branda F, Cañezo VC Jr, Cue EG, Prisno Iii DEL. Artificial intelligence and machine learning approaches for patient safety in complex surgery: a review. Patient Saf Surg. 2025;19:33. 10.1186/s13037-025-00458-8. 41291876 PMC12648791

[16] Unequal treatment, Washington DC. National Academies; 2003. 10.17226/12875.

[17] Loftus TJ, Tighe PJ, Filiberto AC, Efron PA, Brakenridge SC, Mohr AM, Rashidi P, Upchurch GR Jr, Bihorac A. Artificial intelligence and surgical decision-making. JAMA Surg. 2020;155:148–58. 10.1001/jamasurg.2019.4917. 31825465 PMC7286802

[18] ACS NSQIP Participant Use Data File | ACS. American College of Surgeons 2024. (accessed June 12, 2026). https://www.facs.org/quality-programs/data-and-registries/acs-nsqip/participant-use-data-file/. (accessed June 12, 2026).

[19] Halamka J, Bydon M, Cerrato P, Bhagra A. Addressing racial disparities in surgical care with machine learning. NPJ Digit Med. 2022;5:152. 10.1038/s41746-022-00695-6. 36180724 PMC9525720

[20] Vyas DA, Eisenstein LG, Jones DS. Hidden in plain sight - reconsidering the use of race correction in clinical algorithms. N Engl J Med. 2020;383:874–82. 10.1056/NEJMms2004740. 32853499

[21] Hopkins BS, Mazmudar A, Driscoll C, Svet M, Goergen J, Kelsten M, Shlobin NA, Kesavabhotla K, Smith ZA, Dahdaleh NS. Using artificial intelligence (AI) to predict postoperative surgical site infection: A retrospective cohort of 4046 posterior spinal fusions. Clin Neurol Neurosurg. 2020;192:105718. 10.1016/j.clineuro.2020.105718. 32065943

[22] Gichoya JW, Banerjee I, Bhimireddy AR, Burns JL, Celi LA, Chen L-C, Correa R, Dullerud N, Ghassemi M, Huang S-C, Kuo P-C, Lungren MP, Palmer LJ, Price BJ, Purkayastha S, Pyrros AT, Oakden-Rayner L, Okechukwu C, Seyyed-Kalantari L, Trivedi H, Wang R, Zaiman Z, Zhang H. AI recognition of patient race in medical imaging: a modelling study. Lancet Digit Health. 2022;4:e406–14. 10.1016/S2589-7500(22)00063-2. 35568690 PMC9650160

[23] Ghabisha S, Alyhari Q, Ateik A, Al-Wageeh S, Ahmed F, Al-Shehari M, Obadiel Y, Edrees WH, Al-Shehari WA. Machine learning prediction model for surgical site infections after major abdominal surgery. Patient Saf Surg. 2026;20. 10.1186/s13037-026-00481-3. PMC13126701 41840617

[24] Tikkanen RS, Woolhandler S, Himmelstein DU, Kressin NR, Hanchate A, Lin M-Y, McCormick D, Lasser KE. Hospital payer and racial/ethnic mix at private academic medical centers in Boston and New York City. Int J Health Serv. 2017;47:460–76. 10.1177/0020731416689549. 28152644 PMC6090544

[25] Overview of the National (Nationwide). Inpatient Sample (NIS). Agency for healthcare research and quality 2023. https://hcup-us.ahrq.gov/nisoverview.jsp. (accessed June 12, 2026).

[26] Johnson AEW, Bulgarelli L, Shen L, Gayles A, Shammout A, Horng S, Pollard TJ, Moody B, Gow B, Lehman L wei, Celi H, Mark LA. RG. MIMIC-IV, a freely accessible electronic health record dataset. Sci Data 2023;10. 10.1038/S41597-022-01899-X. PMC9810617 36596836

[27] National Joint Registry Annual Report 2025 - HQIP. Healthcare quality improvement partnership 2025. (accessed June 12, 2026). https://www.hqip.org.uk/news/njr_annual_report25/. (accessed June 12, 2026).

[28] Number of cancer cases for selected cancers in the SEER-medicare data. National Cancer Institute 2023. (accessed June 12, 2026). https://healthcaredelivery.cancer.gov/seermedicare/aboutdata/cases.html. (accessed June 12, 2026).

[29] Johnson JA, Moore B, Hwang EK, Hickner A, Yeo H. The accuracy of race & ethnicity data in US based healthcare databases: A systematic review. Am J Surg. 2023;226:463–70. 10.1016/j.amjsurg.2023.05.011. 37230870

[30] Inaccuracies in medicare’s race and ethnicity data hinder the ability to assess health disparities. | Office of Inspector General | Government Oversight | U.S. Department of Health and Human Services. US Department of Health and Human Services 2022. https://oig.hhs.gov/reports/all/2022/inaccuracies-in-medicares-race-and-ethnicity-data-hinder-the-ability-to-assess-health-disparities/. (accessed June 12, 2026).

[31] Smith MC, Ben-Shlomo Y, Dieppe P, Beswick AD, Adebajo AO, Wilkinson JM, Blom AW. National Joint Registry for England W and NI. Rates of hip and knee joint replacement amongst different ethnic groups in England: an analysis of National Joint Registry data. Osteoarthritis Cartilage. 2017;25:448–54. 10.1016/j.joca.2016.12.030. 28159557

[32] Nyawambi TM, Muchiri H. Mitigating racial algorithmic bias in healthcare artificial intelligent systems: a fairness-aware machine learning approach. 2024 5th International Conference on Smart Sensors and Application: Shaping the Future of Intelligent Innovation, ICSSA 2024. 2024. 10.1109/ICSSA62312.2024.10788666.

[33] Yuan Y, Tamo J, Ben, Shi W, Zhong Y, Nnamdi MC, Brenn BR, Hwang SW, Wang MD. Developing fairness-aware task decomposition to improve equity in post-spinal fusion complication prediction. BCB 2025 - Proceedings of the 16th ACM International Conference on Bioinformatics, Computational Biology, and Health Informatics. 2025;1:10. 10.1145/3765612.3767246;WGROUP:STRING:ACM.

[34] Pleiss G, Raghavan M, Wu F, Kleinberg J, Weinberger KQ. On Fairness and Calibration. Adv Neural Inf Process Syst. 2017;2017–December:5681–90. 10.1145/3603195.3603207.

[35] Chouldechova A. Fair prediction with disparate impact: A study of bias in recidivism prediction instruments. Big Data. 2017;5:153–63. 10.1089/big.2016.0047. 28632438

[36] Almarie B, Gonzalez-Gonzalez LF, Dos Santos Barbosa LA, Lutz A, Grosse U, Fregni F. Machine learning-enabled medical devices authorized by the US Food and Drug Administration in 2024: Regulatory characteristics, predicate lineage, and transparency reporting. Biomedicines. 2025;13:3005. 10.3390/biomedicines13123005. 41463017 PMC12730494

[37] Regulation - EU – 2024/1689 - EN - EUR-Lex. European Union 2024. https://eur-lex.europa.eu/legal-content/EN/TXT/?uri=OJ:L_202401689 (accessed June 12, 2026).

[38] Artificial Intelligence. The future is what we make it. Am Coll Surgons 2024;159. 10.1001/JAMASURG.2023.6687.

[39] Allareddy V, Oubaidin M, Rampa S, Venugopalan SR, Elnagar MH, Yadav S, Lee MK. Call for algorithmic fairness to mitigate amplification of racial biases in artificial intelligence models used in orthodontics and craniofacial health. Orthod Craniofac Res. 2023;26(Suppl 1):124–30. 10.1111/ocr.12721. 37846615

[40] Rieke N, Hancox J, Li W, Milletarì F, Roth HR, Albarqouni S, Bakas S, Galtier MN, Landman BA, Maier-Hein K, Ourselin S, Sheller M, Summers RM, Trask A, Xu D, Baust M, Cardoso MJ. The future of digital health with federated learning. NPJ Digit Med. 2020;3:119. 10.1038/s41746-020-00323-1. 33015372 PMC7490367

